# Altered Regulation of adipomiR Editing with Aging

**DOI:** 10.3390/ijms21186899

**Published:** 2020-09-20

**Authors:** Sabel Meadows, Abbagael Seidler, Madison Wall, Jamika Page, Cara Taylor, Brendin Flinn, Robin Turner, Nalini Santanam

**Affiliations:** Department of Biomedical Sciences, Joan C Edwards School of Medicine, Marshall University, Huntington, WV 25755, USA; meadows244@marshall.edu (S.M.); seidler7@marshall.edu (A.S.); wall42@live.marshall.edu (M.W.); jamika.page@towerhealth.org (J.P.); taylor693@live.marshall.edu (C.T.); flinn9@live.marshall.edu (B.F.); roberts2@marshall.edu (R.T.)

**Keywords:** non-coding RNA, miRNA editing, adipose dysfunction

## Abstract

Adipose dysfunction with aging increases risk to insulin resistance and other chronic metabolic diseases. We previously showed functional changes in microRNAs involved in pre-adipocyte differentiation with aging resulting in adipose dysfunction. However, the mechanisms leading to this dysfunction in microRNAs in adipose tissue (adipomiRs) during aging are not well understood. We determined the longitudinal changes in expression of adipomiRs and studied their regulatory mechanisms, such as miRNA biogenesis and editing, in an aging rodent model, with Fischer344 × Brown-Norway hybrid rats at ages ranging from 3 to 30 months (male/females, n > 8). Expression of adipomiRs and their edited forms were determined by small-RNA sequencing. RT-qPCR was used to measure the mRNA expression of biogenesis and editing enzymes. Sanger sequencing was used to validate editing with aging. Differential expression of adipomiRs involved in adipocyte differentiation and insulin signaling was altered with aging. Sex- and age-specific changes in edited adipomiRs were observed. An increase in miRNA biogenesis and editing enzymes (ADARs and their splice variants) were observed with increasing age, more so in female than male rats. The adipose dysfunction observed with age is attributed to differences in editing of adipomiRs, suggesting a novel regulatory pathway in aging.

## 1. Introduction

Aging is a complex process that involves continuous accumulation of cellular damage. Interventions to prevent these damages will improve aging-related pathologies. Adipose tissue dysfunction increases with age, which dramatically alters fat mass, its redistribution and function [1,2,3], leading to metabolic imbalance and increased risk to cardiometabolic diseases. Adipose tissue, the fat-storing endocrine organ, secretes ‘adipokines’ that play a major role in lipid metabolism and insulin sensitivity [4]. Mechanisms leading to functional changes in adipose tissue with age are not fully understood, however, genetic or epigenetic regulation is thought to play a role.

Adipose tissue is made up of heterogeneous populations of cells, which includes mature adipocytes as well as the stromal-vascular fraction consisting of endothelial cells, immune cells, pre-adipocytes and others. The progenitor cells, which include pre-adipocytes and the adipose-derived stem cells within the stromal-vascular fraction, are a tiny niche of cells necessary to replenish the mature adipocytes. Our studies have shown that these progenitor cells lose their stemness, i.e., the multi-lineage potential, with aging. We showed that progenitor cells from older rats had lower capacity to differentiate into adipocytes but retain the capacity to differentiate into osteocytes [5]. This leads to lower availability of mature adipocytes required for maintaining lipid and glucose homeostasis. We further showed that this loss of stemness observed in aging pre-adipocytes was attributed to the dysfunction of key microRNAs essential for adipocyte plasticity. MicroRNAs (miRNA) are endogenously expressed non-coding RNAs and important regulators of gene expression in mammals [6,7]. These master regulators are a cluster of 17–24 nucleotide (nt) single-stranded RNA, that are post-transcriptional regulators of gene expression [6,7]. In the simplest explanation, miRNA synthesis begins with the hairpin structure, primary miRNA “pri-miR”, which is recognized and cleaved by RNA polymerase, Drosha to the smaller 70 nt precursor miRNA “pre-miR”. The pre-miR is then cleaved by Dicer to generate “mature miRNA” [8]. Due to their ability to degrade target mRNA or repress mRNA translation by complementary sequence-specific interactions with their mRNA targets, the miRNAs can modulate biological networks and are therefore sought out extensively as potential risk predictors or therapeutic targets in several diseases (including cancer and cardiometabolic diseases) [9,10,11]. Since miRNAs were originally discovered as a major player in the developmental process, their ability to regulate the aging process was not a surprise [12]. It is consistently observed that miRNA expression is altered in several aging model systems [12,13,14].

There are several studies currently in the literature highlighting differential expression of miRNAs in the adipose tissue (adipomiRs), especially in conditions where it is dysfunctional, such as in cardiometabolic diseases [15,16,17]. MiRNAs involved in adipogenesis were first identified by studying 3T3-L1 adipocyte differentiation and in adipose tissue obtained from high-fat-fed animals [18]. Among the several miRNAs that were altered during adipogenesis, miR-143 was the most investigated and was shown to regulate adipogenesis [18] by modulating its down-stream target, the ERK5 MAP kinase (extracellular signal-regulated kinase5)–peroxisome proliferator-activated receptor γ (PPARγ) pathway [19] and the MAP2K5, a key member of the MAPKK family [20]. Our studies showed that during the differentiation of adipose progenitor cells to mature adipocytes, there was a dysregulation of the key adipogenic miRNAs (miR-143) with aging. This resulted in lower capacity of the progenitor cells from older rats to differentiate into mature adipocytes in contrast to progenitor cells from younger rats. Also, the unresponsiveness of the pre-adipocytes from aging rats to the gain- or loss-of-function of miR-143 further supported the possibility for the role for upstream regulators of miRNA biogenesis or function contributing to the increased pathology seen during aging [5].

Recent advances in the field have identified molecular mechanisms that regulate miRNA biogenesis, maturation and action in a cell-dependent manner, both under physiological and disease conditions. RNA editing is one such post-transcriptional RNA regulatory mechanism that cells and tissues use for generating RNA and protein diversity [21,22]. This phenomenon occurs through post-transcriptional modification of single nucleotides in the pre-mRNA [23,24]. One such type of RNA editing is substitution editing: the conversion of the nucleotide ‘Adenosine (A)’ to ‘Inosine (I)’ in double-stranded (ds) RNA by the “Adenosine Deaminase Acting on RNA (ADAR)” family of enzymes. Mature miRNAs can also undergo ADAR-mediated editing [25]. Earlier studies have shown that RNA editing is compromised during aging [26,27]. There is currently not much known about how adipomiRs are regulated during aging, a knowledge of which will help understand the role of these non-coding RNAs in metabolic diseases associated with aging. In this study, we investigated sex-specific changes in adipomiRs with aging and determined if this dysfunction was due to loss of miRNA biogenesis or due to other regulatory pathways, such as ‘miRNA editing’ [28,29]. Our studies showed sex-specific differences in adipomiRs and its editing with aging. Exploring these novel mechanisms will not only help in understanding the complex cellular role of these non-coding RNAs in metabolic tissues but also help develop better therapeutics to treat adipose-related pathologies during aging.

## 2. Results

### 2.1. Longitudinal Changes in adipomiRs in Each Sex

**Figure 1:**
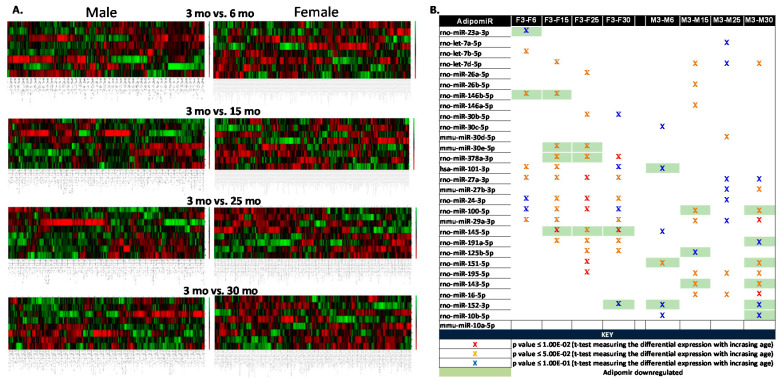
Longitudinal changes in adipose-specific microRNAs (adipomiRs): Small-RNA sequencing was performed on visceral adipose tissue obtained from all ages in both the sexes (*n* = 4/age/sex). (**A**) Heat map depicts the differentially expressed adipomiRs with age (6, 15, 25 and 30 month (mo in the figure) old) compared to 3 month old male and female FBN rats. (**B**) A list of the differentially expressed adipomiRs in both sexes. Green shade indicates downregulation of the respective adipomiR. Red X—*p*-value ≤ 1.00 × 10^−2^; orange X—*p*-value ≤ 5.00 × 10^−2^ and blue X—*p*-value ≤ 1.00 × 10^−1^ (*t*-test measuring the differential expression with increasing age compared to 3 mo rats in both sexes).

MicroRNAs were isolated from the visceral adipose tissue (adipomiRs) collected from FBN rats of all ages (3, 6, 15, 25 and 30 months old, males and females). To find crucial differences in adipomiRs with aging, small-RNA sequencing followed by differential expression analyses was performed by LC Sciences (Houston, TX, USA). A total of 4712 unique miRNAs were determined, of which 565 were known miRNAs identified in *Rattus norvegicus* and 235 miRNAs known in mammals but novel to *Rattus norvegicus*. There were 91 predicted miRNAs mapped to known miRNAs of *Ratttus norvegicus* and genome within hairpins and 1296 mapped to known miRNAs of *Ratttus norvegicus* and genome, no hairpins. 1551 were mapped to mir and miRNAs of *Ratttus norvegicus*, but unmapped to genome, 1144 mapped to known mirs of *Rattus norvegicus* but unmapped to genome. 396 predicted miRNAs were unmapped to known miRNAs but mapped to genome and within hairpins. T-test was used to determine longitudinal changes in adipomiRs using the 3 month old rats in each sex, as the control group. Figure 1A shows the heat map of the differentially expressed adipomiRs in 6, 15, 25 and 30 month old compared to 3 month old rats in males and females. Figure 1B displays adipomiRs that are significantly up- or down-regulated (green shaded) in each of these aging groups compared to 3 month old rats. There were more downregulated adipomiRs (miR-100-5p; miR-191a-5p, miR-151-5p, miR-99b; miR-152-3p; miR-10b-5p; and miR-27b-3p) in 30 month old male rats compared to 3 month old rats.

**Figure 2:**
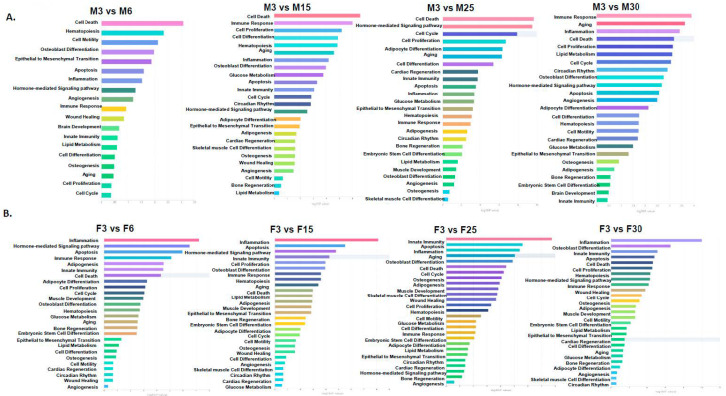
Functional enrichment analysis of the differentially expressed adipomiRs: TAM 2.0 was used to determine microRNA (miRNA)-functional enrichment analysis of adipomiRs that were differentially expressed in each group compared to the 3 mo rat (**A**) Male and (**B**) Female rats. The functions are listed according to the highest to the lowest association with the differentially expressed adipomiRs.

To explore the associated enriched functions of the differentially expressed miRNAs with age in each sex, we used the TAM 2.0 bioinformatics tool to perform functional enrichment analysis. The bar graph in Figure 2 is ranked with the highest (longer bars) enrichment to the lowest (shorter bars) enrichment within each age pair in each sex. As shown in Figure 2A, adipomiRs associated with “inflammation” or “apoptosis” were highly expressed in females, however in male rats, the adipomiRs associated with “cell death” or “immune response” (Figure 2B) were highly expressed. Interestingly, the adipomiRs associated with “aging” were highly expressed in male rats with increasing age compared to female rats.

### 2.2. Differential Expression of adipomiRs and Their Targets Between Two Sexes at Each Age

**Figure 3:**
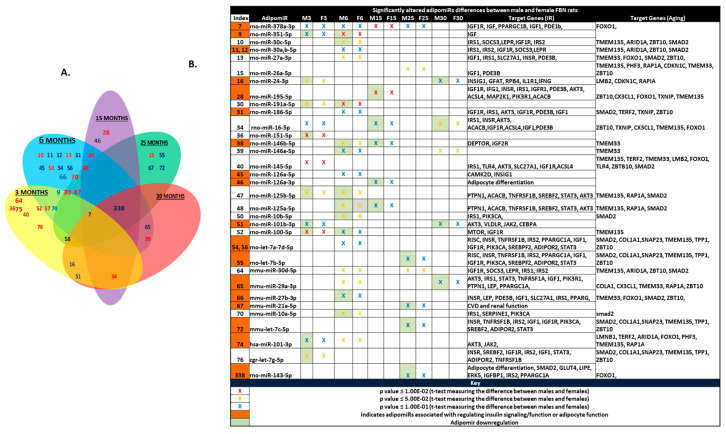
Differential expression of adipomiRs between the two sexes: (**A**) Lotus scheme depicting the differentially expressed adipomiRs in every age group between males and females. Each number in the lotus diagram represents an individual adipomiR whose ID is listed in the accompanying table. Each petal of the lotus scheme shows the adipomiRs that are commonly significantly altered in each group between the two ages. (**B**) Tabular representation of the differentially expressed miRNAs. Targetscan 7.2 was used to identify targets involved in pathways related to insulin resistance and the aging pathway. Orange shaded numbers in the Table represents adipomiRs associated with regulating insulin signaling and/or adipocyte function. Red X—*p*-value ≤ 1.00 × 10^−2^; orange X—*p*-value ≤ 5.00 × 10^−2^ and blue X—*p*-value ≤ 1.00 × 10^−1^ (*t*-test measuring the difference between males and females).

Since we observed longitudinal changes in expression of adipomiRs within each sex with age, we next compared the differential expression of these miRNAs between males and female rats with increasing age. The lotus diagram and the table (Figure 3A,B) show the differentially expressed adipomiRs between the two sexes at each age group (example, M-3 vs. F-3; M-6 vs. F-6). The adipomiRs that were downregulated in one sex compared to the other sex are shown with green shading in Figure 3B. There were several adipomiRs that were downregulated in male rats compared to female rats at all age groups. Downregulation of microRNAs generally results in induction of their target genes.

In order to determine gene targets of these adipomiRs, we ran the Insulin Resistance (IR) RT^2^ Profiler PCR Array (PARN-156Z, Qiagen, Germantown, MD, USA) and Aging RT^2^ Profiler PCR Array (PARN-178Z, Qiagen, Germantown, MD, USA) in rats of all age groups of both sexes (*n* = 4/age/sex). T-tests comparing differential expression between the 3 month old rats and other aging rats within each sex are depicted by red circles (higher expression) and green circles (lower expression) in the network figures generated in Cytoscape 3.7.1 (Supplementary Figure S1A,B). The genes that reached significance within each age group are represented with a black star. There were several IR genes that were upregulated at 15 months of age which were not seen when 6 or 30 month old male rats were compared to 3 month old rats. In contrast, the number of upregulated IR genes was highest in the 30 month old rats compared to 6 or 15 month old female rats. The two genes that were commonly regulated in both males and females with age were phosphoinositide-3 kinase regulator subunit 1 (PiK3r1) (targeted by differentially expressed miR-195-5p, miR-29a-3p, miR-16-5p) which plays an important role in insulin action and is downregulated in IR, and the long-chain fatty acid transport protein, Slc27a1 (targeted by differentially expressed miR-145-5p, miR-27a and b-3p), which is a target of PPARγ, a key transcription factor regulating adipose differentiation and sensitive to senescence. Both of these genes were downregulated with aging in male rats.

There was also a sexual dimorphism in total number of significantly altered aging-related genes, with 32 genes differentially expressed in females compared to 16 genes in male rats. There were several genes that were upregulated in male rats (red circles) with age, such as C1qc (2.6-fold), gfap and lyzl1 (7–8-fold), however the majority of the genes were downregulated in female rats (such as Anxa3, TMEM33, Txnip) with aging (Supplementary Figure S2A,B). There were more significantly altered genes in 6 and 15 month old rats compared to 3 month old female rats, as seen with the black stars.

When Targetscan 7.2 was used to identify gene targets for differentially expressed adipomiRs displayed in Figure 1 and Figure 2, it was interesting to note that several of the target genes were related to IR or aging (Figure 3B). For example, the adipomiR miR-30 family or let-7 family that target either insulin resistance genes (IRS1, IRS2, SOCS3, IGFR2, TNFRSF1B, STAT3, AKT3, PTPN1, ACAB) or genes altered during aging (TMEM 33, TMEM135, ZBT10, SMAD2) were differentially expressed with aging.

### 2.3. Differential Expression of miRNA Biogenesis and Editing Enzymes

**Figure 4:**
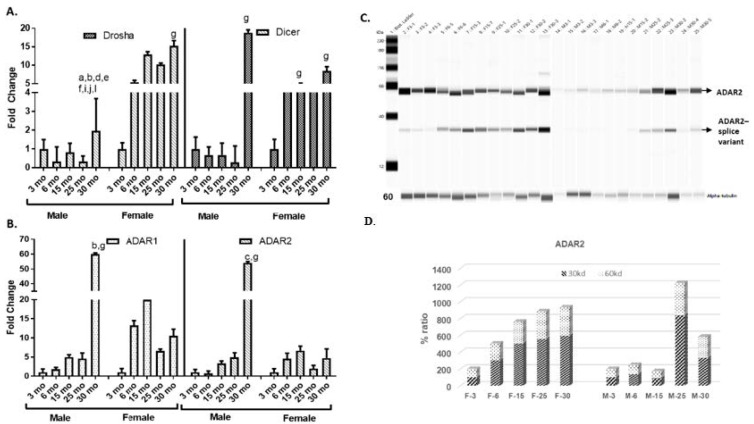
Expression levels of microRNA biogenesis and editing enzymes: (**A**) mRNA expression of Drosha and Dicer, the two enzymes involved in microRNA biogenesis determined in the visceral adipose tissue, showed higher expression in female rats compared to males. (**B**) mRNA expression of editing enzymes, Adenosine Deaminase Acting on RNA (ADAR1 and ADAR2), showed increasing levels with age in male rats. The female rats had higher expression of ADAR1 compared to ADAR2. (**C**) Protein expression of ADAR2 (60 kd) showed higher expression with age in both the sexes, but more prominent in female rats. (**D**) There was increased splice variant (30 kd) expression with age in both sexes. One-way analysis of variance (ANOVA) followed by post-hoc Tukey’s test. ^a^ *p* < 0.05 compared to M-3; ^b^ *p* < 0.001 compared M-6; ^c^ *p* < 0.05 compared M-6; ^d^ *p* < 0.001 compared to M-15; ^e^ *p* < 0.001 compared M-25; ^f^ *p* < 0.001 compared to F-3; ^g^ *p* < 0.05 compared to F-3; ^i^ *p* < 0.001 compared to F-6; ^j^ *p* < 0.001 compared to F-15; ^k^ *p* < 0.05 compared to F-15; ^l^ *p* < 0.05 compared to F-25.

Differences in expression of adipomiRs with age (or sex) might be attributed either to differences in their biogenesis or due to their altered regulation. As seen in Figure 4A, miRNA biogenesis enzymes’ Drosha and Dicer expression, determined in the adipose tissue, showed a 2–3-fold higher expression in female rats compared to male rats. One-way analysis of variance (ANOVA) followed by Tukey’s test showed that the expression of Drosha in 30 month old males was significantly increased compared to females at age 3 mo (*p* < 0.0001), but lower than 6 and 15 months (*p* < 0.0001), and at 25 months (*p* < 0.0092) of age. Drosha expression within males at 30 months of age was increased compared to 3 months (*p* = 0.0114), 6 months (0.0007), 15 months (0.0003) and 25 months (*p* < 0.0001) of age. Female rats at 30 months old also had significantly higher Drosha expression compared to 3 month old rats (*p* = 0.0136). Dicer expression in males at 30 months old were significantly increased compared to females at 3 months (*p* = 0.0135). Within the female rats, the dicer expression was higher in 15 month (*p* = 0.0378) and 30 month (*p* = 0.0324) old rats compared to 3 month old rats.

One of the recently identified pathways by which miRNA expression and activity can be regulated is by undergoing sequence editing through the action of enzymes, such as Adenosine Deaminase Acting on RNA (ADAR1 and ADAR2). ADAR1 and ADAR2 mRNA expression in adipose tissue in all age groups showed increasing levels with age (Figure 4B) within male rats. In contrast, in female rats, the expression in all age groups was at least 3-fold greater than 3 month old rats, however none reached significance. In male rats, ADAR1 expression in 30 month old rats was significantly higher compared to 3 month old male rats (*p* = 0.0462) and female rats (*p* = 0.0116). ADAR2 expression in 30 month old male rats was significantly higher than 6 month old male rats (*p* = 0.0208) or 3 month old female rats (*p* = 0.0472).

The RNA editing enzyme, ADAR1, is ubiquitously present, whereas ADAR2 is inducible in a tissue-specific manner. ADAR2 results in editing of adenosine to inosine (A–I leading to G–C conversion). Hence, we determined if there were any longitudinal changes in protein expression of ADAR2 in adipose tissue at all age groups of rats in both sexes. We measured this using the automated WES system. In contrast to its mRNA expression, there was a longitudinal increase in ADAR2 protein expression (~60 kd) with aging, in both sexes, but specifically in female rats (Figure 4C). In females, there was a significant increase in ADAR2 protein expression in 30 month (*p* = 0.0396) compared to 3 month old rats. In male rats, ADAR2 expression was significantly increased in 25 month old rats compared to 3 month (*p* = 0.0227) and 15 month (*p* = 0.0278) old rats and compared to 3 month (*p* = 0.0213) and 30 month (*p* = 0.0425) old female rats.

Interestingly, we also found an increase in a splice variant of the ADAR2 protein (~30 kd) with age in both female and male rats (Figure 4D). Compared to 3 month old male rats, the splice variant expression was significantly increased in 25 month old male rats (*p* = 0.0167) as well as 25 month (*p* = 0.0454) and 30 month (*p* = 0.0114) old female rats. The expression of the splice variant in 6 and 15 month old male rats was significantly lower than 25 month old male rats (*p* = 0.0460 and *p* = 0.0301, respectively) and 30 month old female rats (*p* = 0.039 and *p* = 0.0245, respectively).

### 2.4. Longitudinal Changes in Edited Adipomirs

**Figure 5:**
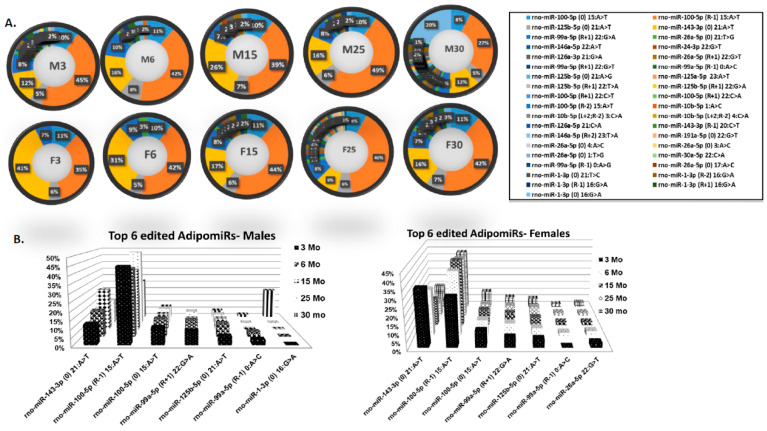
Longitudinal changes in edited adipomiRs: (**A**) Quantitative analysis of the edited adipomiRs from male and female FBN rats as determined by small-RNA sequencing. Each doughnut chart represents the percent of the highly edited adipomiRs in each age group in the two sexes. The larger the slice within the doughnut graph, the larger editing of that individual adipomiR. The table provides the IDs of the adipomiR represented in the slices of the doughnut graphs. (**B**) The graphical representation of the top 6 highly edited adipomiRs at each age group in both the sexes.

AdipomiR expression can differ due to editing or mismatching in the miRNA sequences either within the “seed region” or in the sequences outside the seed regions. We used the small-RNA sequencing data to determine what percent of miRNAs were edited (sequences mismatched) in the adipose tissue from all age groups of rats in both sexes. To do this, we used an arbitrary cut-off of only those miRNAs that had the number of reads > 3000 average of the sequencing dataset and with mismatched sequences from their known mature miRNA sequence. In Figure 5A, the slices within each doughnut show the percent of individual adipomiRs edited in each of the age groups in males and females. In the doughnut figures, the larger the slice of the doughnut, the higher the edited reads within a particular miRNA sequence. As can be seen in the doughnut figures, within both the sexes, the higher the age, the more miRNAs were found edited. Figure 5B represents the top 6 adipomiRs that were found to be edited in each of the age groups in both sexes. Interestingly, the top adipomiRs edited are shown to be involved in adipogenic differentiation (miR-143, miR-125b) or osteogenic differentiation (miR-99a, miR-26a), or both (miR-100 and miR-1). Some of these edited adipomiRs also targeted genes involved in IR or aging (as discussed above).

### 2.5. Validation of rno-miR-143-3p Editing Using Sanger Sequencing

In order to validate miRNA editing during aging, we used the Sanger sequencing technique to determine ADAR2 editing of the mature adipomiR-143-3p. Supplementary Figure S3 displays the edited and un-edited miRNA sequences detected by Sanger sequencing of mature miR-143, at each age group (n > 6 of each age group was run in triplicates). There was very little editing (changes in sequence compared to the original seed sequence of miR-143) within the seed region of miR-143 at all ages in both sexes. Most of the changes in sequences observed were outside the seed region. There seemed to be more editing at younger ages (6 and 15 months) but not in older ages.

## 3. Discussion

Properly functional adipose tissue controls energy balance and has favorable effects on metabolic health and longevity, hence any change in its function leads to metabolic dysfunction. There is a loss of insulin sensitivity, increased redox stress, low-grade inflammation, mitochondrial dysfunction and lipotoxicity [30,31,32]. At present, the mechanisms leading to adipose dysfunction with aging are not completely understood. There exists studies that support the dysfunction of pre-adipocytes with aging, which may be partially due to the accumulation of senescent-associated secretory phenotype, resulting in chronic low-grade inflammation and activation of the JAK pathway [33,34,35]. Recent accumulating evidence also suggests that miRNAs play a key role in adipose tissue formation and function [36,37,38]. Studies have shown differences in expression of miRNAs between various adipose depots as well as brown versus white adipose tissue [39,40]. In this study, we found sexual dimorphism in the differential expression of miRNAs with age in the visceral adipose tissue. Deep sequencing of miRNA isolated from visceral adipose tissue from all age groups showed sex differences in adipomiR expression with age. When miRNAs from 3 month old rats were compared to each of the other age groups (6, 15, 25 and 30 months old), several of the differentially expressed miRNAs targeted genes involved in either miRNA biogenesis (miR-146, Let-7 cluster), adipogenesis (miR-143/145, miR-27b, miR-126a) or insulin signaling (miR-30 and Let-7 cluster). When male and female rats were compared, we found differential expression of miRNAs within each age group (for example, miR-378 and miR-143). The miRNAs differentially expressed decreased with age, especially within the male rats. The differentially expressed adipomiRs, such as miR-143, miR-146, etc., are pro-adipogenic by increasing the expression of transcription factors that are key in adipogenesis, such as PPARγ, GLUT4, adiponectin, etc. [41]. These miRNAs were downregulated in adipose tissue. There are also miRNAs that inhibit adipogenesis but upregulate adipocyte differentiation, such as miR-125 [42] and miR-34a, which target CD36, LXRα, PGC1α, or FASN [42,43]. In addition to adipomiRs, circulating miRNAs such as miR-142, miR-140, miR-138, miR-15a, b and miR-376a are also suggested to be predictive markers for adipose dysfunction [44,45]. A recent study showed significantly elevated levels of circulating miR-143-3p in patients with metabolic syndrome, which, when inhibited in a mouse model, inhibited insulin resistance (IR), and its key target was insulin-like growth factor 2 receptor [16]. Our studies show that the loss of adipogenesis with aging correlated to changes in these miRNAs. Another adipomiR, miR-378, targets MAPK and promotes adipogenesis [46], but also is enriched in brown adipose tissue and promotes differentiation of adipocytes to brown fat by targeting PPARGC1B and the phosphodiesterase, PDE1b, which catalyzes the turnover of cAMP and cGMP [47]. Activation of miR-30b/miR-378 by omega-3 lipids upregulates cAMP and promotes brown adipogenesis [48]. Interestingly, there was an induction of both these adipomiRs with age (25 and 30 months old) in female and not in male FBN rats. Similarly, miRNAs such as the let-7 family or miR-125 a, b regulate TMEM135 [49], ZBT10 and SMAD2 [50], genes that play a role in mitochondrial or ER stress and are altered during aging. Functional enrichment analysis (TAM 2.0) showed that adipomiRs affecting “aging” and “immune response” had the highest hit in male rats, whereas in females, it was mostly affecting “inflammation” and “apoptosis”. Though the two sexes had similar hits on adipomiRs affecting “inflammatory pathways”, the males had a higher hit on the “aging” pathway, as also seen by upregulation of aging-related genes (Supplementary Figure S2A,B) in males.

Changes in the expression of miRNA can be attributed to its altered biogenesis. Drosha and Dicer are key enzymes involved in miRNA biogenesis. It is known that Dicer and miRNA biogenesis are essential for the adipogenic differentiation and not osteogenic differentiation of the adipose-derived stem cells [51], however, this function is independent of Dicer’s role in preventing growth senescence of primary cells [36]. In the present study, there was a sexual dimorphism in the expression levels of Drosha and Dicer with aging. In male rats, the oldest group (30 months) had a higher expression, but the rest of the groups had lower expression levels of these enzymes. However, in females, there was an upregulation of these enzymes at 6 months, after which it remained plateaued at higher age groups. What we observed in male rats was similar to the previously shown lower expression of miRNAs in the adipose tissue with aging in C57Bl6/6J mice (3, 6 and 24 months of age), which was attributed to a lower Dicer expression in the adipose tissue, demonstrating that there might be changes in miRNA processing in adipose tissue during aging [52]. Similarly, the increase in the oldest age (30 months) in male rats (equivalent to humans in their eighth decade) observed in our study might be similar to the observed increase in Dicer expression seen in centenarians compared to octogenarians [53]. However, no such major changes with age were observed in females. There is also evidence for a role for Dicer in insulin/glucose sensitivity. Using Dicer knockout mice, it was shown that circulating exosomal adipomiRs can modulate miRNA expression in other tissues, such as liver, thereby regulating glucose tolerance and insulin sensitivity [54]. Fat-specific Dicer knockout mice exhibited accelerated age-associated insulin resistance and premature mortality [55]. This probably may be why the females had lesser induction of IR-related genes compared to male rats, since they maintained higher Dicer levels. However, in addition to miRNA biogenesis enzymes, other pathways may also play a role in miRNA regulation, such as miRNA editing.

ADARs (ADAR 1–3) catalyze the deamination of adenosine (A) to inosine (I), which results in the inosine being recognized as guanosine (G) during translation. This A–G transformation leads to either mis-sense altered amino acids within the encoded protein during translation or the resulting ‘G-U’ wobble pairs increases risk for degradation [56]. The original function of ADARs was thought to be its re-coding capacity, however it was discovered it also targets non-coding regions and, hence, numerous ADAR-miRNA pathways were identified [29,57]. We also observed sexual dimorphism in the expression levels of ADAR1 and 2. In males, there was a gradual increase in the levels of these enzymes with the oldest (30 months) rats having the highest expression. Whereas, the expression levels of both the enzymes increased at 6 months of age and this was maintained at all older age groups compared to 3 month old rats. Since ADAR2 is inducible, we only determined its protein expression, which showed a gradual increase in expression with age in female rats. Again, in males, the increase in expression was seen more in older rats. These differences may be attributed to sex-hormones regulating ADAR expression and activity. There are earlier studies in Drosophila [58] and human brains [59] where sex-dependent differences in overall ADAR-mediated editing have been observed.

An increase in ADAR expression may reflect in miRNA editing. To explore if any ADAR-mediated editing was seen in our samples, we either used the small-RNA sequencing data to explore for global editing in all adipomiRs that were sequenced, or used Sanger sequencing to detect editing in one of the most commonly studied miRNA that modulates adipocyte differentiation, i.e., miR-143. The next-generation sequencing studies showed editing in the sequences of several adipomiRs in both males and females, however the top six adipomiRs were involved in adipogenic differentiation (miR-143, miR-125b) or osteogenic differentiation (miR-99a, miR-26a), or both (miR-100 and miR-1). Sanger sequencing of the miR-143, one of the key adipomiRs involved in adipocyte differentiation, mostly showed editing outside the “seed region”. Since the majority of the RNA editing regions occur within the intronic or UTR regions, the functional significance of these events is difficult to infer. Computational studies have suggested that RNA editing within the 3′UTR are under functional constraint in order to avoid the interruption of miRNA target sites [60]. However, some RNA editing events might edit functional miRNA target sites. The target recognition sites of miRNAs are located at nucleotides positions 2–7 nt or “miRNA seed regions” [61]. Single-base substitutions in the “seed regions” might lead to miRNA variants whose target gene specificity might be drastically different from its unedited miRNA [62]. This redirection of the target gene specificity might be beneficial as well as detrimental. For example, less A–I editing of the miR-376 cluster promoted migration and invasiveness in glioblastoma cells. The genes targeted by the edited or unedited miR-376 clusters had varying potential to either suppress or promote invasiveness [63]. Hence, editing of miR-143 outside the seed region may not have directly affected its targets. But, editing of the other adipomiRs might result in miRNA variants with varying target specificity.

The editing activity of the ADARs is fine-tuned by its alternate splicing events. Both ADAR1 and ADAR2 undergo alternate splicing, resulting in several putative isoforms. ADAR2 undergoes two distinct alternate splicing events, at the 5′ end of the coding sequence, resulting in the inclusion of 47 nucleotides, leading to a truncated inactive protein (ADAR2e and ADAR2f) with 82 amino acids in the rodents and 31 amino acids in humans. The second alternate splicing involves the 3′ coding sequence (catalytic domain) and results in the exclusion of 30 nucleotides in rodents and 120 nucleotides in humans, resulting in ADAR2a (presence of the cassette) and ADAR2b (absence of the cassette) isoforms [64,65]. This second type of splicing results in loss of catalytic activity. Interestingly, we observed a splice variant (~30 kd protein) with aging in both male and female rats. It is possible that the truncated protein that we found in our aging rats might be the one where there is loss of catalytic activity. RNA editing is compromised during aging [26]. In humans, several single nucleotide polymorphisms in the RNA editing genes ADARB1 and ADARB2 were seen in extremely old age in a US-based study on centenarians. The presence of SNPs in ADARs increased mortality in these centenarians [66]. Hence, changes in ADAR activity in metabolic tissues during aging will result in risk of metabolic diseases.

Overall, our studies showed that there was sex-dependent differential expression of adipomiRs with aging. This variation in adipomiRs might be attributed to differences in expression and activity of RNA editing enzymes such as ADAR2. Future studies should explore editing in other adipomiRs that are important in the adipose dysfunction. Our findings thus far are significant in that it points to the possibility of additional miRNA regulatory mechanisms that can lead to changes in metabolic changes. In addition to the miRNA biogenesis regulation previously studied in aging, the ADAR-mediated editing mechanisms may also contribute as additional checkpoints in aging and aging-related disorders.

## 4. Materials and Methods

### 4.1. Animals

The National Institutes of Aging (NIA, Bethesda, MD, USA)-approved Fischer 344 x Brown Norway hybrid rats (FBN) (Charles River, Wilmington, MA, USA) were used in our studies. Probability of survival curves provided for the FBN hybrid rats by the NIA were employed to select age groups corresponding roughly to humans in their teenage (3 month old rats), third (6 month old rats), fourth (14 month old rats), sixth (24 month old rats) and eighth (30 month old rats) decade of life [67]. We chose this latter time point in view of the fact that this age group represents one of the fastest growing segments of the aging population in the USA [68]. After one week of acclimatization, male and female FBN rats (*n* = 8/age group/sex), were fasted overnight and then were sacrificed. After sacrifice, gonadal and retroperitoneal fat depots were excised. Visceral abdominal fat from the intra-abdominal region (gonadal) was used for all the studies. All animal studies were performed after approval by Marshall University’s Institutional Animal Care and Use Committee. All ethical procedures were used.

### 4.2. RNA (miRNA) Extraction

Total RNA (including miRNA) was isolated from visceral adipose tissue (100 mg) using the miRNeasy Mini Kit (Qiagen, Germantown, MD, USA) under cold conditions, following the manufacturer’s protocol. The concentration of the isolated RNA that contains miRNA and its quality were analyzed by the Nanodrop model 1000 (Thermo Scientific, Nanodrop Technologies Inc, Waltham, MA, USA) and its integrity was determined on a 1.2% agarose gel electrophoresis. Only RNA with a RIN number > 7 (Agilent Bioanalyzer, Santa Clara, CA, USA) was used for the small-RNA deep-sequencing and RT^2^ PCR array analyses.

### 4.3. Differential Expression of adipomiRs and Edited adipomiR Detection using Small-RNA Sequencing

The small-RNA sequencing and differential expression of adipomiRs were performed (in biological quadruplicates and experimental triplicates for each age group of each sex) using the Illumina TruSeq approach by LC Sciences, LLC (Houston, TX, USA). (A) Small-RNA Library Construction: A small RNA library was generated using the Illumina Truseq^TM^ Small-RNA Preparation kit according to Illumina’s TruSeq^TM^ Small-RNA Sample Preparation Guide. (B) Deep Sequencing: The purified cDNA library was used for cluster generation on Illumina’s Cluster Station and then sequenced on the Illumina HiSeq platform. Raw sequencing reads (50 nt) were obtained using Illumina’s Sequencing Control Studio software version 2.8 (SCS v2.8) following real-time sequencing image analysis and base-calling by Illumina’s Real-Time Analysis version 1.8.70 (RTA v1.8.70). (C) Bioinformatics Analysis: A proprietary pipeline script, ACGT101-miR v4.2 (LC Sciences, Houston, TX, USA), was used for sequencing data analysis [69,70,71]. The single end-sequencing reads were cleaned with quality filter, adapter cutter and length filter. The cleaned reads were mapped to miRBase. Normalization of sequence counts in each sample was achieved by dividing sequence counts of individual samples with corresponding normalization factors, which are the median values of the ratios between specific sample counts and geometric mean counts of all samples. For differential expression analysis of adipomiRs, *t*-test was used for “between groups” comparison to the 3 month age group. (D) Target and functional prediction: Targetscan 7.2 (Whitehead Institute, Cambridge, MA, USA) was used to identify target genes for the differentially expressed adipomiRs. TAM 2.0 (Hebei University, Tianjin and Peking University, Bejing, China) was used for miRNA functional enrichment analysis. We chose the overrepresentation analysis function of TAM 2.0 [72] that helped assess the up and down miRNAs involved in various functions. (E) Detection of edited miRNA: For detection of edited adipomiRs, the in-depth sequencing data were analyzed in silico to detect the adipomiRs with sequencing reads > 3000 and had mismatched sequences. The total percent of each of the edited adipomiRs were represented as individual slices in a doughnut chart. The top 6 edited adipomiRs in each age-group and sex were represented graphically.

### 4.4. Aging and Insulin Resistance RT^2^ Profiler PCR Array

To detect target genes of the differentially expressed adipomiRs, we used PCR arrays to detect key genes involved in either “insulin resistance” or “aging”. The changes in “insulin resistance”- and “aging”-related genes were performed on total RNA extracted from visceral adipose tissue of all four age groups (3, 6, 15 and 30 months old) for female/male FBN rats. The genes assayed in 3 month old rats for both sexes were defined as control (CTRL). 1 μg of purified RNA was used for amplification to cDNA.

The rat Insulin Resistance RT^2^ Profiler PCR Array (PARN-156Z, Qiagen, Germantown, MD, USA) profiles the expression of 84 genes involved in insulin and adipokine signaling, genes commonly dysregulated in type 2 diabetes, genes involved in innate immunity and inflammatory processes and enzymes and transporters important for carbohydrate and lipid metabolism.

The rat Aging RT^2^ Profiler PCR Array (PARN-178Z, Qiagen, Germantown, MD, USA) that profiles the expression of 84 genes related to ‘Aging’ was performed on all samples. The aging array included genes involved in apoptosis, cell cycle, cell senescence, inflammation and mitochondrial function.

The 384-well plate array was performed using the Roche 480 Real-Time PCR system (Roche, Indianapolis, IN, USA) following the manufacturer’s instructions. The data obtained was interpreted using the Qiagen PCR Array data analysis web portal available online on the manufacturer’s website. Quality control of all the PCR arrays was measured by assessing the quality of internal controls, such as RTC (Reverse Transcription Control) and GPC (Genomic DNA Contamination Control). An array with the RTC value ≤ 5 and GPC value ≥ 35 passes the quality control. The array design and final data processing were consistent with the requirements of Minimum Information about a Microarray Experiment [73].

The variations between groups were defined as fold changes in gene expression in aged animals compared to gene expression in the younger (3 month old) animals in the two sexes. Cytoscape 3.7.1 [74] was used to perform pathway analysis of the differentially and significantly expressed genes in all age groups compared to 3 month old rats. Cytoscape software was also used to determine pathway analysis of the differentially expressed genes within each age group between the two sexes.

### 4.5. MiRNA Biogenesis and Editing Enzyme Expression

The expression levels of miRNAs can be regulated by altering their biogenesis or by editing their sequences. The mRNA expression of the enzymes involved in miRNA biogenesis (Drosha and Dicer) and those involved in miRNA editing (ADAR1 and ADAR2) were performed using RT-qPCR. cDNA was synthesized from 1 µg of total RNA isolated from visceral adipose tissue from all age groups of FBN rats, of both sexes, using the iScript cDNA synthesis kit (170-8890, Bio-Rad, Hercules, CA, USA). Real-time PCR was carried out in 25 μL of a SYBR green reaction mixture containing 1 μL of cDNA iQSYBR Green Supermix (170-8882, Bio-Rad, Hercules, CA, USA), and the respective primers in duplicates. The following primers were used: Drosha: 5-ctccccgcctaccaactttt-3 and 3-attggtcagaggggcatgtg-5; Dicer: 5-gaagaggagaccagcgttcc-3 and 3-cgggtttggggtaactctcc-5; ADAR1: 5-agttgggggccgctggtttc-3 and 3-tgcatccgctgcgtcttgct-5; ADAR2-ADARB1: 5-cgctggaacgtggtgggcat-3, 3-ccctggagaggtggtcccg-5. 18s was used as the housekeeping gene. 18s: 5′-gcaattattccccatgaacg-3′, 3′-ggcctcactaaaccatccaa-5′. Fold change was calculated using the Pfaffl equation [75]. The 3 month old rat from each sex was used as the control group.

### 4.6. Detection of ADAR Protein Using WES

Protein levels of ADAR2 were determined using the WES (automated Western Blotting system) system (Protein Simple, San Jose, CA, USA) according to the manufacturer’s instructions using the 12–230 kDa separation module and anti-mouse detection module (Protein Simple, San Jose, CA, USA). Briefly, 3 µg of adipose protein lysate was diluted in 10× sample buffer and mixed with Fluorescent master mix (all provided in the manufacturer’s kits). This sample was heated at 95 °C for 5 min. The heated samples, standards, blocking reagent (antibody diluent), 1:25 diluted primary antibody (Anti-ADAR2, sc-73409, Santa Cruz Biotechnology, Inc. Dallas, TX, USA), secondary antibody (provided in the kit) and chemiluminescent substrate were pipetted into the separate module and run on the Protein Simple WES system. Alpha-tubulin (1:100) was used as the house-keeping protein. Densitometry of the protein bands were determined using the Compass for Simple Western software (Protein Simple, San Jose, CA, USA). The ratio of the ADAR2 band to the alpha-tubulin for each of the groups were averaged and presented, graphically.

### 4.7. Sanger Sequencing for Detecting ADAR-Edited Sequences in miR-143

MiRNA isolated from adipose tissue from all groups of rats (n > 6/age) was subjected to Taqman PCR by first using RT primer of rno-miR-143 followed by Taq PCR using Taq primer of miR-143 (Qiagen, Germantown, MD, USA). The Taq PCR product of the miR-143 was cloned into pRC2.1-TOPO cloning vector that contains M13 (Thermonth Fisher Scientific, Waltham, MA, USA). The cloned products were transformed into “One shot chemically competent *E. coli* bacteria”. Three or more bacterial colonies from each rat sample were cultured in LB media and plasmid DNA was prepared. The plasmid DNA preparations were then sent to Marshall University Genomics Core facility for Sanger Sequencing. After Sanger sequencing, the sequences from each animal were uploaded into commercially available programs (CLC Workbench, Qiagen, Redwood City, CA, USA and BioEdit 7.2, Ibis Biosciences, Carlsbad, CA, USA) for alignment to the miR-143 sequence. A–I to G–C changes in the sequence (most common nucleotide editing due to ADARs) were determined in all Sanger sequences of miR-143 sequenced from all age groups of rats in both sexes.

### 4.8. Statistics

For the Rat RT^2^ Profiler PCR array, the statistical significance in fold changes between young (3 months) and all other age groups (6, 15, 25 and 30 months) for each sex was automatically generated by the Qiagen PCR array online data analysis portal. Fold changes > 3.0 or < 0.3 were defined as fold increase or decrease and used for statistical analysis. The significant differences between the ΔCt values between groups were analyzed using two-tailed Student’s *t*-test. For the RT-qPCR analysis, one-way analysis of variance (ANOVA) was performed at the level of ΔCt, in order to exclude potential bias due to averaging of data transformed through the Pfaffl equation 2^−(ΔΔCt)^ [75]. Significance was confirmed using post-hoc analysis with Tukey’s test.

## Supplementary Materials

Supplementary materials can be found at https://www.mdpi.com/1422-0067/21/18/6899/s1.

## References

[1] Toth M.J., Tchernof A. (2000). Lipid metabolism in the elderly. Eur. J. Clin. Nutr..

[2] Ghosh A.K., O’Brien M., Mau T., Qi N., Yung R. (2019). Adipose Tissue Senescence and Inflammation in Aging is Reversed by the Young Milieu. J. Gerontol. Biol. Sci. Med. Sci..

[3] Zoico E., Rubele S., De Caro A., Nori N., Mazzali G., Fantin F., Rossi A., Zamboni M. (2019). Brown and Beige Adipose Tissue and Aging. Front. Endocrinol..

[4] Lago F., Dieguez C., Gómez-Reino J., Gualillo O. (2007). Adipokines as emerging mediators of immune response and inflammation. Nat. Clin. Pract. Rheumatol..

[5] Fei J., Tamski H., Cook C., Santanam N. (2013). MicroRNA regulation of adipose derived stem cells in aging rats. PLoS ONE.

[6] Davis B.N., Hata A. (2009). Regulation of MicroRNA Biogenesis: A miRiad of mechanisms. Cell Commun. Signal..

[7] Bartel D.P. (2004). MicroRNAs: Genomics, biogenesis, mechanism, and function. Cell.

[8] Reinhart B.J. (2002). Small RNAs Correspond to Centromere Heterochromatic Repeats. Science..

[9] Wang Y., Liang Y., Lu Q. (2008). MicroRNA epigenetic alterations: Predicting biomarkers and therapeutic targets in human diseases. Clin. Genet..

[10] Tsai L.M., Yu D. (2010). MicroRNAs in common diseases and potential therapeutic applications. Clin. Exp. Pharmacol. Physiol..

[11] Mishra P.K., Tyagi N., Kumar M., Tyagi S.C. (2009). MicroRNAs as a therapeutic target for cardiovascular diseases. J. Cell. Mol. Med..

[12] Bates D.J., Liang R.-Q., Li N., Wang E. (2009). The impact of noncoding RNA on the biochemical and molecular mechanisms of aging. Biochim. Biophys. Acta (BBA) Gen. Subj..

[13] Liang R.-Q., Bates D.J., Wang E. (2009). Epigenetic Control of MicroRNA Expression and Aging. Curr. Genom..

[14] Lafferty-Whyte K., Cairney C.J., Jamieson N.B., Oien K.A., Keith W.N. (2009). Pathway analysis of senescence-associated miRNA targets reveals common processes to different senescence induction mechanisms. Biochim. et Biophys. Acta (BBA) Mol. Basis Dis..

[15] Kilic I.D., Dodurga Y., Uludag B., Alihanoglu Y.I., Yildiz B.S., Enli Y., Secme M., Bostancı H.E., Yildiz B.S. (2015). microRNA -143 and -223 in obesity. Gene.

[16] Lin X., Tang S., Gui W., Matro E., Tao T., Li L., Wu F., Zhou J., Zheng F., Li H. (2019). Circulating miR-143-3p inhibition protects against insulin resistance in Metabolic Syndrome via targeting of the insulin-like growth factor 2 receptor. Transl. Res..

[17] Li B., Fan J., Chen N. (2018). A Novel Regulator of Type II Diabetes: MicroRNA-143. Trends Endocrinol. Metab..

[18] Esau C., Kang X., Peralta E., Hanson E., Marcusson E.G., Ravichandran L.V., Sun Y., Koo S., Perera R.J., Jain R. (2004). MicroRNA-143 Regulates Adipocyte Differentiation. J. Boil. Chem..

[19] Takanabe R., Ono K., Abe Y., Takaya T., Horie T., Wada H., Kita T., Satoh N., Shimatsu A., Hasegawa K. (2008). Up-regulated expression of microRNA-143 in association with obesity in adipose tissue of mice fed high-fat diet. Biochem. Biophys. Res. Commun..

[20] Chen L., Hou J., Ye L., Chen Y., Cui J., Tian W., Li C., Liu L. (2014). MicroRNA-143 Regulates Adipogenesis by Modulating the MAP2K5–ERK5 Signaling. Sci. Rep..

[21] Zamyatnin A.A., Lyamzaev K.G., Zinovkin R.A. (2010). A-to-I RNA editing: A contribution to diversity of the transcriptome and an organism’s development. Biochemestry.

[22] Maas S., Kawahara Y., Tamburro K.M., Nishikura K. (2006). A-to-I RNA editing and human disease. RNA Boil..

[23] Maas S. (2010). Gene regulation through RNA editing. Discov. Med..

[24] Eisenberg E., Li J.B., Levanon E.Y. (2010). Sequence based identification of RNA editing sites. RNA Boil..

[25] Blow M.J., Grocock R.J., Van Dongen S., Enright A.J., Dicks E., Futreal P.A., Wooster R., Stratton M.R. (2006). RNA editing of human microRNAs. Genome Boil..

[26] Montano M., Long K. (2011). RNA surveillance—An emerging role for RNA regulatory networks in aging. Ageing Res. Rev..

[27] Nicholas A., De Magalhães J.P., Kraytsberg Y., Richfield E., Levanon E.Y., Khrapko K. (2010). Age-related gene-specific changes of A-to-I mRNA editing in the human brain. Mech. Ageing Dev..

[28] Yang Y., Zhou X., Jin Y. (2013). ADAR-mediated RNA editing in non-coding RNA sequences. Sci. China Life Sci..

[29] Tomaselli S., Bonamassa B., Alisi A., Nobili V., Locatelli F., Gallo A. (2013). ADAR Enzyme and miRNA Story: A Nucleotide that Can Make the Difference. Int. J. Mol. Sci..

[30] Fei J., Cook C., Blough E., Santanam N. (2010). Age and sex mediated changes in epicardial fat adipokines. Atherosclerosis.

[31] Sepe A., Tchkonia T., Thomou T., Zamboni M., Kirkland J.L. (2010). Aging and regional differences in fat cell progenitors – A mini-review. Gerontology.

[32] Xu M., Palmer A.K., Ding H., Weivoda M.M., Pirtskhalava T., A White T., Sepe A., O Johnson K., Stout M.B., Giorgadze N. (2015). Targeting senescent cells enhances adipogenesis and metabolic function in old age. eLife.

[33] Stout M.B., Justice J.N., Nicklas B.J., Kirkland J.L. (2017). Physiological Aging: Links Among Adipose Tissue Dysfunction, Diabetes, and Frailty. Physiology.

[34] Zhu Y., Tchkonia T., Stout M.B., Giorgadze N., Wang L., Li P.W., Heppelmann C.J., Bouloumié A., Jensen M.D., Bergen H.R. (2015). Inflammation and the depot-specific secretome of human preadipocytes. Obesity.

[35] Xu M., Tchkonia T., Ding H., Ogrodnik M., Lubbers E., Pirtskhalava T., White T.A., Johnson K.O., Stout M.B., Mezera V. (2015). JAK inhibition alleviates the cellular senescence-associated secretory phenotype and frailty in old age. Proc. Natl. Acad. Sci. USA.

[36] Mudhasani R., Imbalzano A.N., Jones S.N. (2010). An essential role for Dicer in adipocyte differentiation. J. Cell. Biochem..

[37] Mudhasani R., Puri V., Hoover K., Czech M.P., Imbalzano A.N., Jones S.N. (2011). Dicer is required for the formation of white but not brown adipose tissue. J. Cell. Physiol..

[38] Kim H.-J., Cho H., Alexander R., Patterson H.C., Gu M., Lo K.A., Xu D., Goh V.J., Nguyen L.N., Chai X. (2014). MicroRNAs Are Required for the Feature Maintenance and Differentiation of Brown Adipocytes. Diabetes.

[39] Icli B., Feinberg M.W. (2017). MicroRNAs in dysfunctional adipose tissue: Cardiovascular implications. Cardiovasc. Res..

[40] Klöting N., Berthold S., Kovács P., Schön M.P., Fasshauer M., Ruschke K., Stumvoll M., Blüher M. (2009). MicroRNA Expression in Human Omental and Subcutaneous Adipose Tissue. PLoS ONE.

[41] Xie H., Lim B., Lodish H.F. (2009). MicroRNAs Induced During Adipogenesis that Accelerate Fat Cell Development Are Downregulated in Obesity. Diabetes.

[42] Rockstroh D., Löffler D., Kiess W., Landgraf K., Körner A. (2016). Regulation of human adipogenesis by miR125b-5p. Adipocyte.

[43] Lavery C.A., Kurowska-Stolarska M., Holmes W.M., Donnelly I., Caslake M., Collier A., Baker A.H., Miller A.M. (2016). miR-34a(-/-) mice are susceptible to diet-induced obesity. Obesity.

[44] Zhang T., Brinkley T.E., Liu K., Feng X., Marsh A.P., Kritchevsky S., Zhou X., Nicklas B.J. (2017). Circulating MiRNAs as biomarkers of gait speed responses to aerobic exercise training in obese older adults. Aging.

[45] Pescador N., Pérez-Barba M., Ibarra J.M., Anchuelo A.C., Martínez-Larrad M.T., Serrano-Ríos M. (2013). Serum Circulating microRNA Profiling for Identification of Potential Type 2 Diabetes and Obesity Biomarkers. PLoS ONE.

[46] Huang N., Wang J., Xie W., Lyu Q., Wu J., He J., Qiu W., Xu N., Zhang Y. (2015). MiR-378a-3p enhances adipogenesis by targeting mitogen-activated protein kinase 1. Biochem. Biophys. Res. Commun..

[47] Pan D., Mao C., Quattrochi B., Friedline R.H., Zhu L.J., Jung D.Y., Kim J.K., Lewis B., Wang Y.-X. (2014). MicroRNA-378 controls classical brown fat expansion to counteract obesity. Nat. Commun..

[48] Kim J., Okla M., Erickson A., Carr T., Natarajan S.K., Chung S. (2016). Eicosapentaenoic Acid Potentiates Brown Thermogenesis through FFAR4-dependent Up-regulation of miR-30b and miR-378. J. Biol. Chem..

[49] Exil V., Avila D.S., Benedetto A., Exil E.A., Adams M.R., Au C., Aschner M. (2010). Stressed-Induced TMEM135 Protein Is Part of a Conserved Genetic Network Involved in Fat Storage and Longevity Regulation in Caenorhabditis elegans. PLoS ONE.

[50] Blumensatt M., Wronkowitz N., Wiza C., Cramer A., Mueller H., Rabelink M.J., Van Weerden W., Eckel J., Sell H., Ouwens D.M. (2014). Adipocyte-derived factors impair insulin signaling in differentiated human vascular smooth muscle cells via the upregulation of miR-143. Biochim. et Biophys. Acta (BBA) Mol. Basis Dis..

[51] Martin E.C., Qureshi A.T., Llamas C.B., Burow M.E., King A.G., Lee O.C., Dasa V., Freitas M.A., Forsberg J.A., Elster E.A. (2018). Mirna biogenesis pathway is differentially regulated during adipose derived stromal/stem cell differentiation. Adipocyte.

[52] Mori M.A., Raghavan P., Thomou T., Boucher J., Robida-Stubbs S., Macotela Y., Russell S.J., Kirkland J.L., Blackwell T.K., Kahn C.R. (2012). Role of MicroRNA Processing in Adipose Tissue in Stress Defense and Longevity. Cell Metab..

[53] Borrás C., Serna E., Gambini J., Inglés M., Vina J. (2017). Centenarians maintain miRNA biogenesis pathway while it is impaired in octogenarians. Mech. Ageing Dev..

[54] Thomou T., Mori M.A., Dreyfuss J.M., Konishi M., Sakaguchi M., Wolfrum C., Rao T.N., Winnay J.N., Garcia-Martin R., Grinspoon S.K. (2017). Adipose-Derived Circulating miRNAs Regulate Gene Expression in Other Tissues. Nature.

[55] Reis F.C.G., Brandão B.B., Branquinho L.O., Guerra B.A., Silva I.D., Frontini A., Thomou T., Sartini L., Cinti S., Kahn C.R. (2016). Fat-specific Dicer deficiency accelerates aging and mitigates several effects of dietary restriction in mice. Aging.

[56] Gott J.M., Emeson R.B. (2000). Functions and Mechanisms of RNA Editing. Annu. Rev. Genet..

[57] Nishikura K. (2006). Editor meets silencer: Crosstalk between RNA editing and RNA interference. Nat. Rev. Mol. Cell Boil..

[58] Jepson J.E., Savva Y.A., Yokose C., Sugden A.U., Sahin A., Reenan R.A. (2011). Engineered alterations in RNA editing modulate complex behavior in Drosophila: Regulatory diversity of adenosine deaminase acting on RNA (ADAR) targets. J. Biol. Chem..

[59] Silvestris D.A., Picardi E., Cesarini V., Fosso B., Mangraviti N., Massimi L., Martini M., Pesole G., Locatelli F., Gallo A. (2019). Dynamic inosinome profiles reveal novel patient stratification and gender-specific differences in glioblastoma. Genome Biol..

[60] Liang H., Landweber L.F. (2007). Hypothesis: RNA editing of microRNA target sites in humans?. RNA.

[61] Friedman R.C., Farh K.K.-H., Burge C.B., Bartel B. (2008). Most mammalian mRNAs are conserved targets of microRNAs. Genome Res..

[62] Kawahara Y., Zinshteyn B., Sethupathy P., Iizasa H., Hatzigeorgiou A.G., Nishikura K. (2007). Redirection of Silencing Targets by Adenosine-to-Inosine Editing of miRNAs. Science.

[63] Choudhury Y., Tay F.C., Lam D.H., Sandanaraj E., Tang C., Ang B.-T., Wang S. (2012). Attenuated adenosine-to-inosine editing of microRNA-376a* promotes invasiveness of glioblastoma cells. J. Clin. Investig..

[64] Rueter S.M., Dawson T.R., Emeson R.B. (1999). Regulation of alternative splicing by RNA editing. Nature.

[65] Feng Y., Sansam C.L., Singh M., Emeson R.B. (2006). Altered RNA Editing in Mice Lacking ADAR2 Autoregulation. Mol. Cell. Biol..

[66] Sebastiani P., Montano M., Puca A., Solovieff N., Kojima T., Wang M.C., Melista E., Meltzer M., Fischer S.E.J., Andersen S.L. (2009). RNA Editing Genes Associated with Extreme Old Age in Humans and with Lifespan in C. elegans. PLoS ONE.

[67] Turturro A., Witt W.W., Lewis S., Hass B.S., Lipman R.D., Hart R.W. (1999). Growth curves and survival characteristics of the animals used in the Biomarkers of Aging Program. J. Gerontol. Ser. A: Biol. Sci. Med. Sci..

[68] Lunenfeld B. (2008). An Aging World—Demographics and challenges. Gynecol. Endocrinol..

[69] Li M., Xia Y., Gu Y., Zhang K., Lang Q., Chen L., Guan J., Luo Z., Chen H., Li Y. (2010). MicroRNAome of Porcine Pre- and Postnatal Development. PLoS ONE.

[70] Wei Z. (2011). Novel and Conserved Micrornas in Dalian Purple Urchin (Strongylocentrotus Nudus) Identified by Next Generation Sequencing. Int. J. Biol. Sci..

[71] Meyer C., Grey F., Kreklywich C.N., Andoh T.F., Tirabassi R.S., Orloff S.L., Streblow D.N. (2010). Cytomegalovirus MicroRNA Expression Is Tissue Specific and Is Associated with Persistence. J. Virol..

[72] Li J., Han X., Wan Y., Zhang S., Zhao Y., Fan R., Cui Q., Zhou Y. (2018). TAM 2.0: Tool for MicroRNA set analysis. Nucleic Acids Res..

[73] Brazma A., Hingamp P., Quackenbush J., Sherlock G., Spellman P., Stoeckert C., Aach J., Ansorge W., Ball C.A., Causton H.C. (2001). Minimum information about a microarray experiment (MIAME)—toward standards for microarray data. Nat. Genet..

[74] Shannon P., Markiel A., Ozier O., Baliga N.S., Wang J.T., Ramage D., Amin N., Schwikowski B., Ideker T. (2003). Cytoscape: A Software Environment for Integrated Models of Biomolecular Interaction Networks. Genome Res..

[75] Pfaffl M.W. (2001). A new mathematical model for relative quantification in real-time RT-PCR. Nucleic Acids Res..

