# Supplementary material for: Altered Regulation of adipomiR Editing with Aging
Source: Int J Mol Sci. 2020 Sep 20;21(18):6899. doi: 10.3390/ijms21186899 (PMC7555933; doi:10.3390/ijms21186899)
Supplement: Supplementary file 1 [file ijms-21-06899-s001.pdf]

SUPPLEMENTARY FIGURES:

Supplementary Figure 1

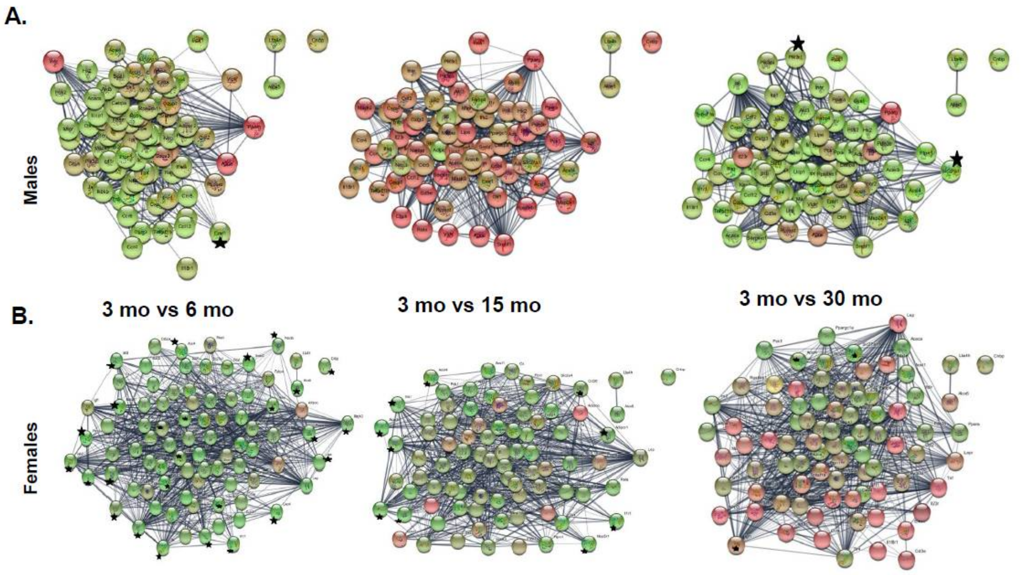

Supplementary Figure 1: Longitudinal changes in Insulin Resistance genes: Target genes for the differentially expressed adipomiRs were determined using PCR array. Genes involved in insulin resistance within the adipose tissue were determined using rat Insulin Resistance RT^2 Profiler PCR Array in rats of all age groups of **A.** Males and **B.** Females (n=4/age/sex). There expression of IR related genes were higher in 15 mo male rats but more so in 30 mo female rats as shown by the red circles (higher expression) and green circles (lower expression) and significant genes with a black star in the network figures generated in Cytoscape 3.7.1.

Supplementary Figure 2

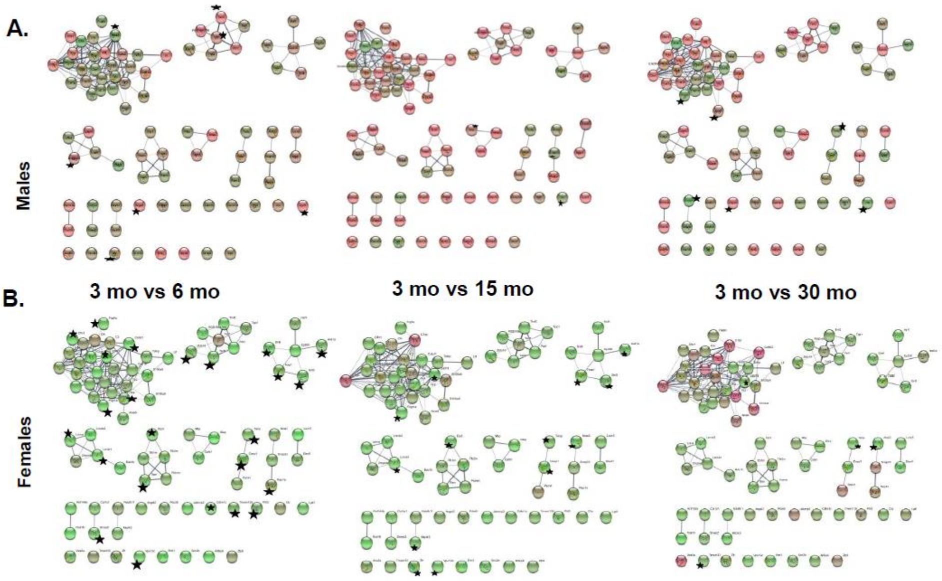

Supplementary Figure 2: Longitudinal changes in Aging related genes in adipose tissue: Target genes for the differentially expressed adipomiRs were determined using PCR array. Genes involved in aging or senescence were determined using RT^2 Profiler PCR Array in **A.** Males and **B.** Females. There were several genes that were up-regulated in male rats (red circles) with age, however

majority of the genes were down-regulated in female rates with aging and significance represented using black stars.

Supplementary Figure 3

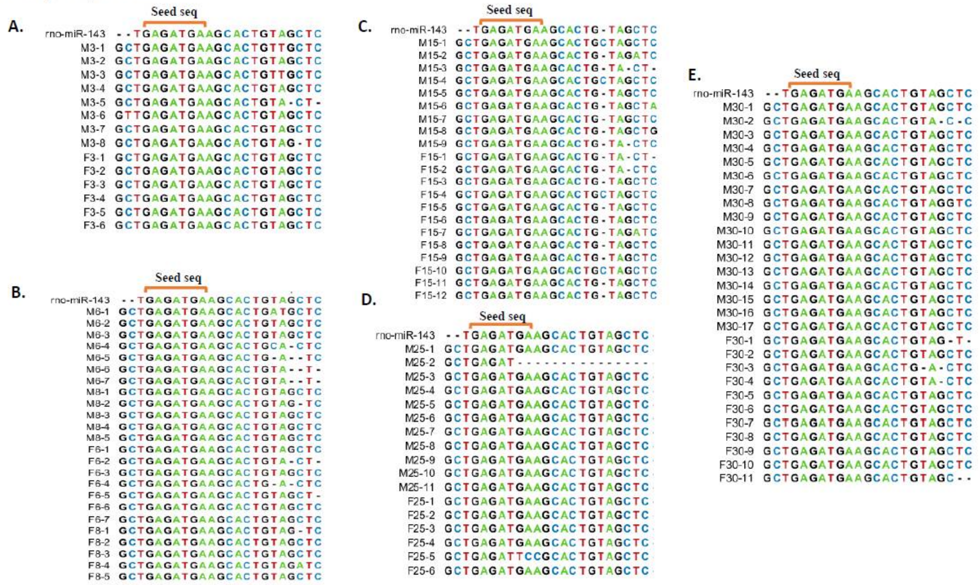

Supplementary Figure 3: Sanger sequence of miR-143 in different ages: Analysis of chromatograms from Sanger sequencing of mature miR-143 in visceral adipose tissue from all age groups of both the sexes. Sequences were compared to the known sequence of the mature rno-miR-143. Any mismatches or editing within the sequences were determined. Majority of the ADAR edited sequences was observed outside the "seed" sequence of miR-143.
